# Secreting-lux/pT-ClyA engineered bacteria suppresses tumor growth via interleukin-1β in two pathways

**DOI:** 10.1186/s13568-019-0910-6

**Published:** 2019-11-21

**Authors:** Yuqin Wu, Zhicai Feng, Shengnan Jiang, Jing Chen, Yuefu Zhan, Jianqiang Chen

**Affiliations:** 1 Department of Radiology, Affiliated Haikou Hospital of Xiangya Medical College, Central South University, No. 43, Renmin Avenue, Haikou, 570208 Hainan China; 2 Department of Oncology, The Xiangya Hospital, Central South University, Changsha, 410013 Hunan China; 3 Department of Radiology, Hainan Maternal and Children’s Medical Center, No. 7, Haifu Road, Haikou, 570206 Hainan China

**Keywords:** Engineered *Salmonella typhimurium*, IL-1β, TLR4, NLRP3, Colon cancer

## Abstract

Engineered *Salmonella typhimurium* (*S.t*-ΔpG^lux/pT-ClyA^) and attenuated *Salmonella typhimurium* (SL: *Salmonella typhimurium* with a defect in the synthesis of guanine 5′-diphosphate-3′-diphosphate) exhibit similar tumor targeting capabilities (Kim et al. in Theranostics 5:1328–1342, 2015; Jiang et al. in Mol Ther 18:635–642, 2013), but *S.t*-ΔpG^lux/pT-ClyA^ exerts superior tumor suppressive effects. The aim of this study was to investigate whether *S.t*-ΔpG^lux/pT-ClyA^ inhibits colon cancer growth and recurrence by promoting increased IL-1β production. The CT26 tumor mouse model was used, and mice were treated in the following ways: PBS, *S.t*-ΔpG^lux/pT-ClyA(+)^ + IL-1βAb, SL, *S.t*-ΔpG^lux/pT-ClyA(−)^, and *S.t*-ΔpG^lux/pT-ClyA(+)^. Dynamic evaluation of the efficacy of *S.t*-ΔpG^lux/pT-ClyA^ in the treatment of colon cancer was assessed by MRI. Western blot, immunofluorescence and flow cytometry analysis were used to investigate IL-1β-derived cells and IL-1β expression on tumor cells and immune cells to analyze the regulatory mechanism. IL-1β levels in tumors colonized by *S.t*-ΔpG^lux/pT-ClyA^ were significantly increased and maintained at high levels compared to control treatments. This increase caused tumors to subside without recurrence. We examined the immune cells mediating *S.t*-ΔpG^lux/pT-ClyA^-induced tumor suppression and examined the major cell types producing IL-1β. We found that macrophages and dendritic cells were the primary IL-1β producers. Inhibition of IL-1β in mice treated with *S.t*-ΔpG^lux/pT-ClyA^ using an IL-1β antibody caused tumor growth to resume. This suggests that IL-1β plays an important role in the treatment of cancer by *S.t*-ΔpG^lux/pT-ClyA^. We found that in *St*-ΔpG^lux/pT-ClyA^-treated tumors, expression of molecules involved in signaling pathways, such as NLRP3, ASC, Caspase1, TLR4, MyD88, NF-kB and IL-1β, were upregulated, while in ΔppGpp *S. typhimurium* treated animals, TLR4, MyD88, NF-kB and IL-1β were upregulated with NLRP3, ASC, and Caspase1 being rarely expressed or not expressed at all. Using *S.t*-ΔpG^lux/pT-ClyA^ may simultaneously activate TLR4 and NLRP3 signaling pathways, which increase IL-1β expression and enhance inhibition of colon cancer growth without tumor recurrence. This study provides a novel platform for treating colon cancer.

## Introduction

Colon cancer is one of the most common malignant tumors in the world. With changing lifestyles and increasing lifespans, the incidence of colon cancer is increasing by the year. In 2018, the estimated number of new cases of colon cancer in the United States was 97,220, and the number of deaths was estimated at 50,630 (Siegel et al. 2018). Bacterial-mediated cancer therapy (BCT) was originally used to treat solid tumors (Nguyen and Min 2017). A variety of bacteria have been found to be useful in anti-tumor therapy, such as *Bifidobacterium* (Luo et al. 2016), *Escherichia* (Ninomiya et al. 2014), *Clostridium* (Luo et al. 2016), *Salmonella* (Park et al. 2016; Nguyen and Min 2017) and *Listeria* (Loessner and Weiss 2004; van Pijkeren et al. 2010). Compared to peripheral tumor proliferative tissues, facultative anaerobic bacteria, such as attenuated *Salmonella* and *Escherichia coli*, are more likely to colonize hypoxic and necrotic areas by accurately targeting the hypoxic regions of solid tumors (Xie et al. 2018; Frahm 2015; Bolhassani and Zahedifard 2012). As conventional therapies fail to provide sustainable remission for most cancer patients, the emerging and unique immune therapeutic approach of bacteria-mediated cancer therapy (BCT) is marching towards a feasible solution.

Attenuated *S. typhimurium* that is defective in ppGpp (ΔppGpp *S. typhimurium*) synthesis has tumor-targeting ability and significantly suppresses tumor growth (Cao et al. 2016; Yoon et al. 2018; Vola et al. 2018). Colonization and subsequent proliferation of ΔppGpp *S. typhimurium* within tumor tissues induces infiltration of immune cells, such as neutrophils, macrophages, and dendritic cells, which then secrete pro-inflammatory cytokines, such as IL-1β, which contribute to anti-cancer efficacy (Yu 2018; Qu et al. 2012; Palsson-McDermott et al. 2015; Kim et al. 2015). The latest literature reports that ΔppGpp *S. typhimurium* exerts anti-cancer effects by promoting secretion of IL-1β from macrophages or dendritic cells. However, this method is typified by tumor recurrence after treatment, which is related with decreased IL-1β levels (Kim et al. 2015). Some studies have shown that low concentrations of IL-1β promote the secretion of IL-17 from CD4+ T cells, inhibiting the body’s anti-tumor mechanisms. High concentrations of IL-1β activate CD8+ T cells, which promotes anti-tumor effects (Ghiringhelli et al. 2009; Bruchard et al. 2013). IL-1β production is closely related to the toll receptor 4 (TLR4) signaling and NOD-like receptor NLRP3 signaling pathways (Jimenez-Dalmaroni et al. 2016; Kim et al. 2015). Lipopolysaccharide (LPS) (Mariathasan et al. 2004) and cytolysin (ClyA) (Wallace et al. 2000) are important ligands for the activation of TLR4 and NLRP3 signaling pathways, respectively. LPS is an endotoxin of gram-negative bacteria (Kahler et al. 2018). The ligand bacterial toxin of NLRP3 can be activated from formed plasma membrane pores, thereby activating NLRP3. However, the pores formed on the cell membrane are rapidly closed after the external stimulus is cleared, so it is difficult to maintain sustained release of IL-1β (Jia et al. 2018). Of note, cytolysin A (ClyA) can act on the mammalian cell membrane to form a persistent pore, resulting in a significant outflow of K^+^ from the cell (Jia et al. 2018), thereby activating the NLRP3 inflammatory body pathway (Gupta et al. 2014). In addition, the combination of attenuated *Salmonella* and ClyA genes through bioengineering technology may offer the possibility sustained, increased release of IL-1β. This may overcome the phenomenon of tumor recurrence caused by downregulation of IL-1β in the tumor microenvironment during the late stage of colon cancer treatment by a single attenuated *Salmonella*.

Previous studies have successfully transfected the ClyA gene into ΔppGpp *S. typhimurium* using genetic engineering technology to form an engineered *Salmonella*: *S.t*-ΔpG^lux/pT-ClyA^, which was confirmed to significantly inhibit subcutaneous inoculation of tumors in CT26 colon cancer (Jiang et al. 2013). To investigate whether *St*-ΔpG^lux/pT-ClyA^ inhibits colon cancer growth by promoting increased production of IL-1β, we dynamically evaluated the efficacy of *S.t*-ΔpG^lux/pT-ClyA^ for colon cancer treatment by MRI and explored the cellular origin of IL-1β in colon cancer treatment. In addition, we assessed the effect of IL-1β on tumors and analyzed the regulatory mechanisms involved using molecular biology techniques.

## Materials and methods

### Tumor cell line and animal model

The murine CT26 colon adenocarcinoma cell line was obtained from the cell bank of the Chinese Academy of Sciences (Shanghai, People’s Republic of China). Cells were grown at 37 °C and 5% CO_2_ in Dulbecco’s Modified Eagle’s Medium (Gibco^®^, Life Technologies, Carlsbad, CA, USA) supplemented with 10% fetal bovine serum and 1% penicillin/streptomycin (HyClone Laboratories, Inc., Logan, UT, USA).

Male *BALB/c* mice (4–5 weeks old) were purchased from the Experimental Animal Center of Central South University and were housed individually at 22 °C to 25 °C under a 12 h light/dark cycle with free access to food and water. To generate in situ colorectal cancer in mice, CT26 cells were injected into the right side of the mouse through a 1 ml syringe to achieve subcutaneous tumor formation. After the tumor body grew to a certain size, subcutaneous tumor-forming mice were euthanized, and the tumor mass was cut into 1–2 mm pieces. The tumor block was then transplanted into the cecum of a normal mouse to establish an orthotopic colon cancer model, as previously described (Rajput et al. 2008). When tumor volume exceeded 2000 mm^3^, animals were euthanized and excluded from the experiment. All animal experimental procedures used in this study were approved by the Animal Ethics Committee of Central South University and conducted in accordance with the Guideline of the Care and Use of Laboratory Animals in Central South University.

### Animal experiments

Tumor-forming mice were treated with PBS, SL, *S.t*-ΔpG^lux/pT-ClyA(+/−)^, or *S.t*-ΔpG^lux/pT-ClyA^ + IL-1βAb. Experimental groups included a placebo (Intravenous injection of PBS, 100 µl), SL treatment (Intravenous injection of 3 × 10^7^ CFU ΔppGpp *S. typhimurium* suspension, 100 µl), and engineered *Salmonella St*-ΔpG^lux/pT-ClyA^ treatment group (Intravenous injection of 3 × 10^7^ CFU *S.t*-ΔpG^lux/pT-ClyA^ suspension, 100 µl). Mice were fed daily doxycycline (17 mg/kg/day) to induce ClyA protein expression (previous studies have achieved relevant results) (Chen et al. 2015; Jiang et al. 2010, 2013). We injected 5 µg of IL-1β antibody (AF401-NA, R&D Systems) into tumor-bearing mice via the tail vein 1 day before and during treatment (two times a week). The mice were euthanized, and tumors were isolated at the end of the experiment. The tumor size of each group was measured using MRI. Tumor volumes (mm^3^) were estimated using the formula (L × H×W)/2 where L is the length, W is the width, and H is the height of the tumor in millimeters (Yu et al. 2004).

### Preparation of engineered *S. typhimurium*

The primers, plasmids, and bacterial strains (*S. typhimurium* ΔppGpp/lux) used in this study were kindly provided by Shengnan Jian (Chonnam National University, South Korea). *S. typhimurium* ΔppGpp/lux-pTet/ClyA(S.t-ΔpG^lux/pT-ClyA^) were constructed as described previously (Jiang et al. 2013; Williams et al. 2010). We constructed the plasmid pJL43 based on the plasmid pJL39. The antitumor gene (ClyA) was placed under the control of the TetR system in the pJL43 plasmid and was then transformed into the *S. typhimurium* ΔppGpp/lux strains. All the primers and constructs used in this study are listed in Tables 1 and 2.

**Table 1:** Primers used in this study

Target	Primer	Template
Tetra	RItetR, 5′-TTAAGACCCACTTTCACATT-3′	TH9952
	RItetA, 5′-CTAAGCACTTGTCTCCTG-3′	
RBS-MCS I	TetPXbaIF, 5′-ACTTTTATCTAATCTAGACATCA-3′	pJL30A
	TetPXbaIrev, 5′-GCCGCCATGGCCCGGGATCCTGCAGGCCTTCTCTATCACTGATAGGGAGT-3′	
RBS-MCS II	TetRNruIF, 5′-TACTAAGTCATCGCGATGGAGCAA-3′	pJL30A
	TerRNruIrev, 5′-AATCCTCGACAGGCTTCTCGAGTGGCCACTCCTGCTTAAGACCCACTTTCACATTTAAGT-3′	
ClyA(A)	ClyAtetAProF, 5′-GTGAAATGACCGGAATATTTGCAGAACAAACT-3′	pAClyA
	ClyAtetAProRev, 5′-ACGCGGATCCTCAGACGTCAGGAACCTCGAA-3′

**Table 2:** Plasmids and *Salmonella typhimurium* strains used in this study

Strains/plasmids	Description
pGEM-T vector	Purchased from Promega
pJL30A	pGEM-T vector encode into the “tetRA” of PCR product
pJL32	Deleted TetA gene of pJL30A and encode “RBS-MCS I” of PCR product under the TetA promoter
pJL37	Encode the ‘RBS-MCS II’ of PCR product into pGEM-T vector
pJL39	pJL32 and pJL37 were digested by Nru I and Sca I restriction enzymes, the construction of pJL39 was ligated by the small fragment of pJL32 and large fragment of pJL37
pJL43 (pT-ClyA) *ΔppGpp* Salmonella typhimurium	pJL39 encode into cytolysin A under TetA promoter *Salmonella typhimurium* is defective in ppGpp
S.t-ΔpGlux	*ΔppGpp* Salmonella typhimurium with the luciferase gene operon luxCDABE
S.t-ΔpGlux/pT-ClyA	pJL43 was transformed into S.t-ΔpGlux

### MRI and optical bioluminescence imaging

Magnetic resonance imaging (MRI) was performed using a 3.0 T MRI system (Signa HDxt; GE Healthcare Bio-Sciences Corp., Piscataway, NJ, USA) with a small animal head coil (Shanghai Chenguang Medical Technologies Co. Ltd, Shanghai, People’s Republic of China). MRI sequences and parameters were as follows: T2-weighted sequence (echo train length = 4; repetition time = 3000 ms; echo time = 120 ms; number of averages = 1) and T1-weighted sequence (repetition time = 350 ms; echo time = minimum value; number of average = 2) before and after injection of 0.2 mL contrast medium (gadodiamide, Omniscan; Amersham Health, Princeton, NJ, USA). Images were acquired for eight slices in the axial plane (slice thickness = 2.0 mm; matrix = 256 × 192). Mice were anesthetized with isoflurane (initial dose 2.5%, maintenance 1.6%). Bioluminescence imaging was performed with the IVIS 100 system (Caliper Life, Sciences, Hopkinton, MA, USA) (Jiang et al. 2013; Nguyen et al. 2010; Massoud and Gambhir 2003). ImageJ was used to measure tumor volume and necrotic volume.

### Western blot analysis, immunofluorescence and antibodies

The third day after bacterial treatment, tumor tissue was taken. After resecting tumors from rats, tumors were homogenized on ice in RIPA buffer (Sigma-Aldrich Co.) containing protease inhibitor cocktail (Sigma-Aldrich Co.). After 120 min of incubation and 30 min of centrifugation (12,000 rpm at 4 °C), the supernatant was then pipetted off and kept on ice. Protein concentrations were measured using the bicinchoninic acid assay kit (Bio-Rad Laboratories Inc., Hercules, CA, USA). Protein samples were subjected to SDS-PAGE and transferred to a nitrocellulose membrane (Bio-Rad). Membranes were probed with polyclonal goat anti-mouse antibodies followed by horseradish peroxidase-conjugated donkey anti-goat IgG (sc-2020, Santa Cruz Biotechnology; 1:2000 dilution). Protein levels were determined using enhanced chemiluminescence plus (Amersham, Buckinghamshire, UK) and Image Reader (LAS-3000 Imaging System; Fuji Photo Film, Tokyo, Japan). Details concerning protein extraction and western blot (WB) can be found in our previous study (Chen et al. 2015).

Immunol Fluorescence was performed using the Immunol Fluorescence Staining Kit with kFluor555-labeled goat anti-rabbit IgG (Keygenbiotech) according to the manufacturer’s instructions. For immunofluorescence staining, tissue sections were permeabilized and blocked in TBS containing 0.1% Tween 20, 0.3% Triton X-100, and 5% BSA. Sections were then incubated with antibodies overnight at 4 °C followed by addition of secondary antibodies (all from Invitrogen). All antibodies were diluted 1:200 in TBS containing 0.1% Tween 20, 1% BSA, and 0.1% Triton X-100. Samples were mounted using Pro-long^®^ Gold antifade reagent (P36930, Invitrogen) and analyzed under a Fluoview-1000 (FV-1000) laser scanning confocal microscope (Olympus). The antibodies used in this article are as follows: NLRP3 (1:1000), ASC (1:1000), Caspase-1 (1:500), IL-1β (1:300), TLR4 (1:1000), MyD88 (1;500), NF-κB (1;300), GAPDH (1:500). Antibodies for NLRP3, ASC and caspase-1 were from Adipogen International (San Diego, CA, USA). Antibody for IL-1β, TLR4 and MyD88 was purchased from R&D Systems (Minneapolis, MN, USA). NF-κB were from Cell Signaling Technology (Beverly, MA, USA). Antibody for GAPDH was from Sigma-Aldrich (St. Louis, MO, USA). Secondary antibodies for western blot were from Sigma-Aldrich (St. Louis, MO, USA). Secondary antibodies for immunofluorescence were from Jackson Immunoresearch Laboratories (West Grove, PA, USA) and Abcam (Cambridge, MA, USA).

### Measurement of IL-1β levels in tumor tissues and serum

Blood was collected by cardiac puncture at 0, 0.5, 3, 12 and 24 h, and then serum was harvested by removing the clotted blood after centrifugation. Cytokine levels were measured using individual ELISA kits (eBioscience) according to the manufacturer’s instructions. The substrate color reaction was measured at 450 nm in an ELISA plate reader (SpectraMax, Molecular Devices).

### Flow cytometry analysis

Mice were euthanized, and spleen tissue was isolated and made into a single cell suspension for flow cytometry analysis. Cells were digested with 0.25% trypsin, centrifuged, and then resuspended in PBS and transferred to an EP tube. Cells were fixed with the addition of 200 µl of 1% paraformaldehyde. Cells were washed with PBS and 200 µl of 90% methanol was added, and cells were then placed at 4 °C. The primary antibody was incubated after washing with PBS. Antibodies used in these experiments include CD11c (N418), CD68 (FA-11), Ly6C (HK1.4), Ly6G (1A8), and IL-1β (NJTEN3). The secondary antibody was incubated after washing with PBS. Flow cytometry was performed using a BD LSR II and BD FACS Aria III flow cytometer (BD Bioscience), and data were analyzed using FlowJo software (TreeStar, Ashland, OR).

### Statistical analysis

GraphPad Prism 7 software was used to analyze data. Survival analysis was performed using a Kaplan–Meier curve. All data are expressed as the mean ± SD, and data were considered significant at p < 0.05.

## Results

### Establishment of BALB/c colon cancer model

**Fig. 1:**
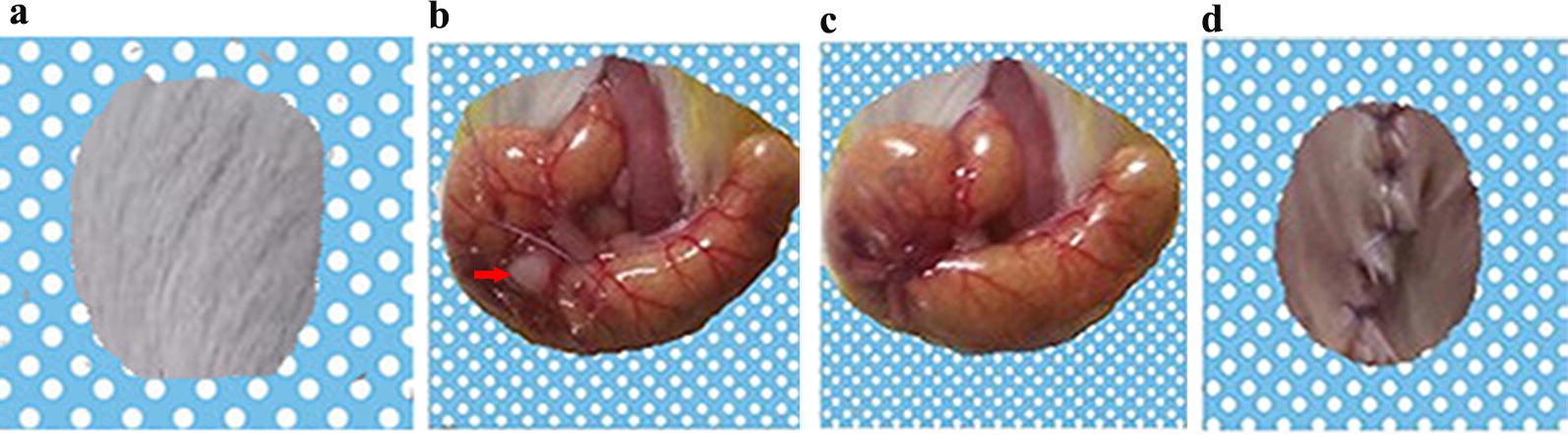
The modeling process. Tumor fragments (approximately 1–2 mm^3^) were transplanted onto the ceca colon. The picture represents the mouse orthotopic colon cancer modeling process, with specific steps from **a** → **d**. A piece of tumor (each measuring 1–2 mm^3^) is indicated with arrow

58 *BALB/c* mice were subjected to orthotopic colon cancer surgery with no deaths occurring during the operation. During the recovery period of 4 days, 8 mice died. On the fifth day after surgery, mice were randomly divided into 5 groups: tumor-bearing mice treated with PBS, SL, *S.t*-ΔpG^lux/pT-ClyA^ ^(+/−Doxycycline induction)^, *S.t*-ΔpG^lux/pT-ClyA^ + IL-1βAb. The modeling process is shown in Fig. 1.

### Lux/pT-ClyA-secreting engineered bacteria to target CT26 tumor mice

**Fig. 2:**
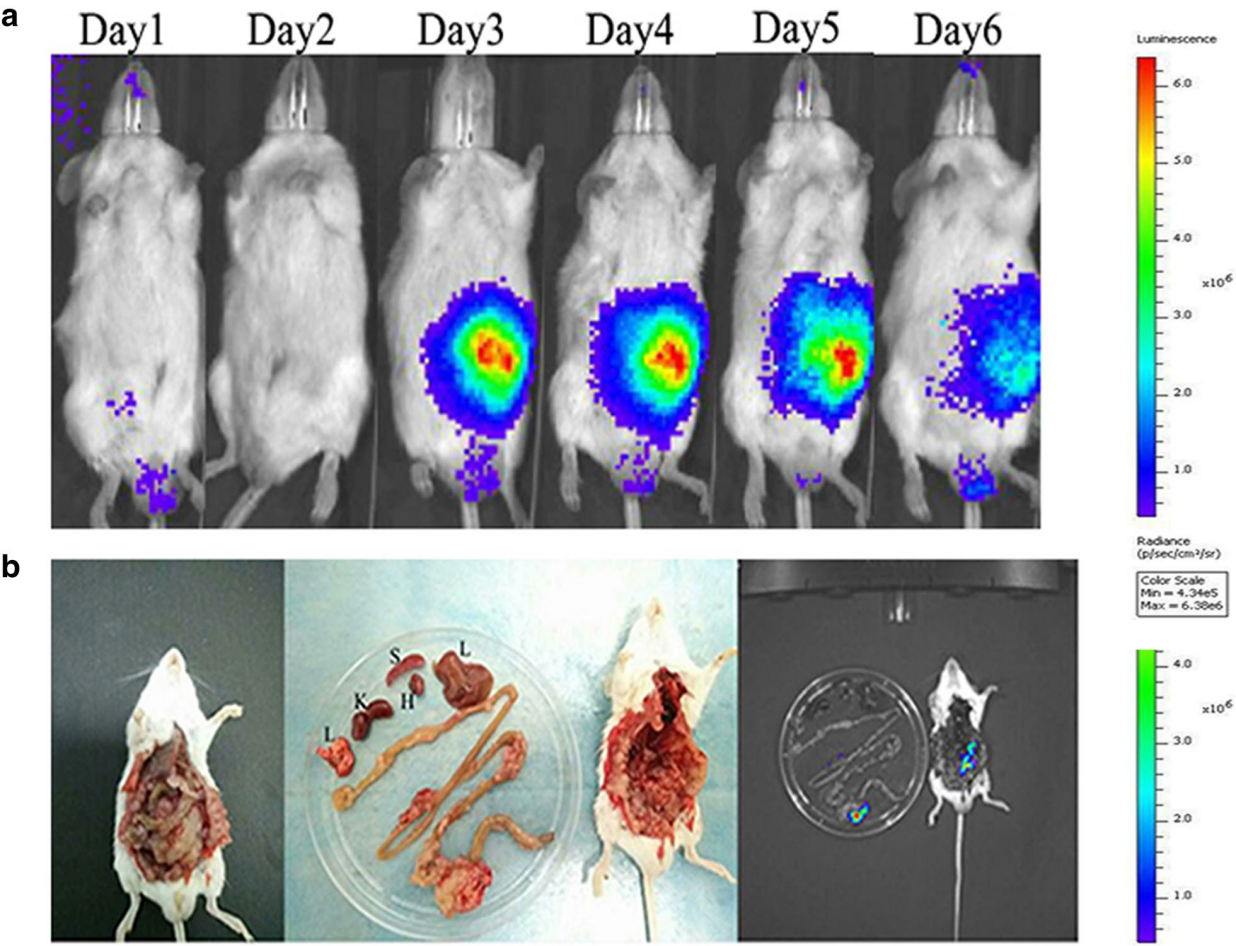
Imaging of lux/pT-ClyA-expressing *S. typhimurium* in a mouse model of cecal colon cancer. *BALB/c* mice were surgically implanted with CT26 tumors, each measuring 1–2 mm^3^, with fragments being transplanted onto the ceca colon. Distribution of bacteria visualized by in vivo bioluminescence imaging after injection of engineered *Salmonella typhimurium* secreting lux/pT-ClyA. Images show representative mice before and after euthanasia. **a** Bioluminescence image shows that bacteria began to colonize the tumor the third day after the injection of the engineered bacteria into the tail vein. **b** On the sixth day, mice were dissected. *L* liver, *S* spleen, *K* kidney, *H* heart, *L* lung (the above images are from the same mouse)

We examined the tumor-targeting activity of *S.t*-ΔpG^lux/pT-ClyA^ in BALB/c mice with CT26 cecal cancer. Bacterial bioluminescence was detected in tumors of injected mice (Fig. 2). These results indicate that the genetically engineered bacteria maintained their ability to target tumors.

### Tumor suppressive effects of engineered bacteria secreting lux/pT-ClyA

**Fig. 3:**
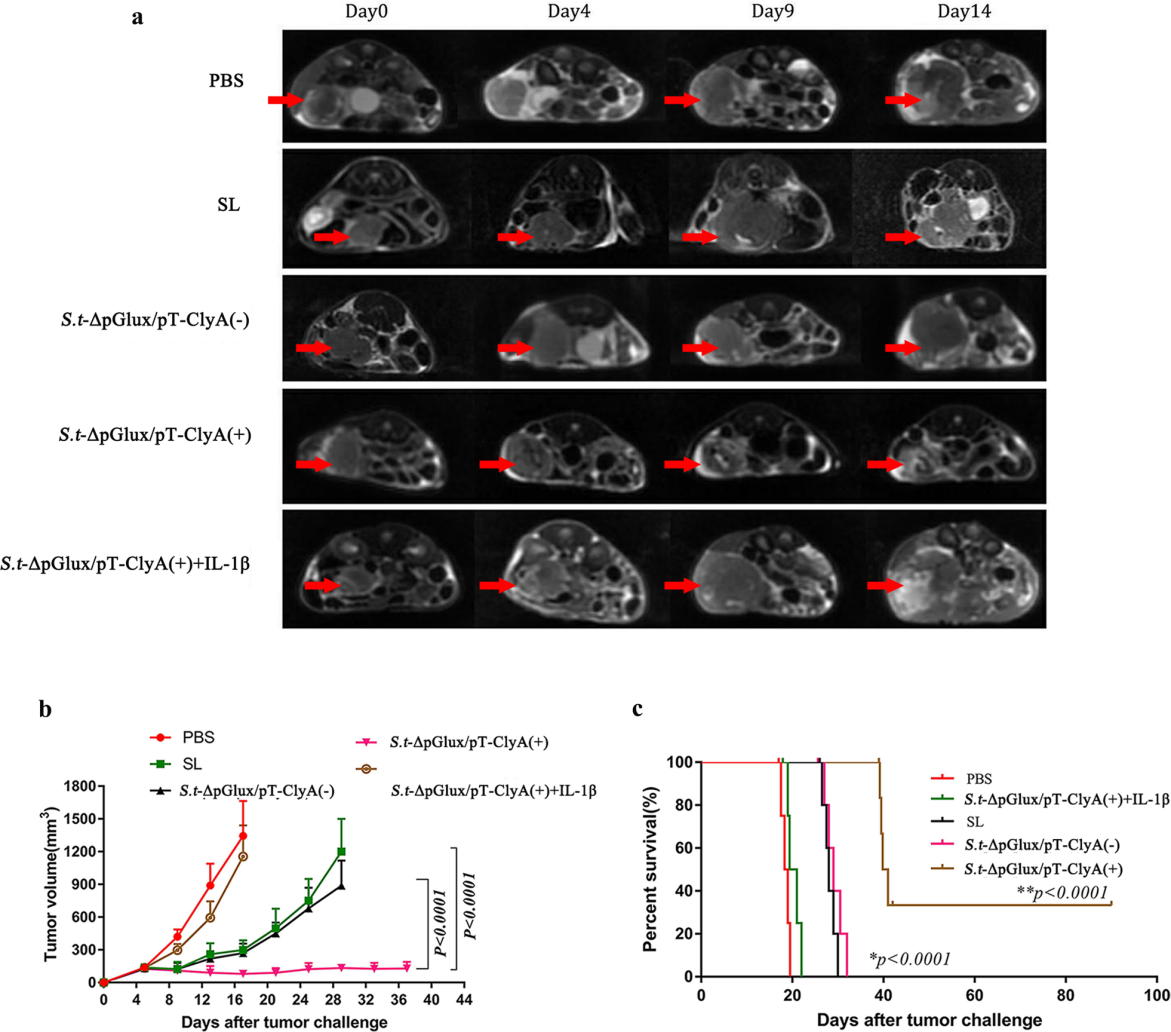
Effect of engineered lux/pT-ClyA-expressing *Salmonella* on growth and survival of CT26 tumors. BALB/c mice (n = 6 per group) were surgically implanted with one piece of CT26 tumor in the ceca colon. When tumors reached a volume of approximately 120–180 mm^3^, mice were divided into five treatment groups. Groups received intravenous (i.v.) injections of PBS, ΔppGpp *S. typhimurium*, or ΔppGpp *S. typhimurium* carrying lux/pT-ClyA either with (+) or without (−) doxycycline induction. For IL-1β depletion, mice received an i.v. injection of anti-IL-1β-specific antibody (IL-1β Ab) 1 day before engineered *Salmonella typhimurium* secreting lux/pT-ClyA (Day 1). The antibody was then injected twice a week for 2 weeks. Where relevant, mice received 17 mg/kg/day doxycycline. **a** Images of tumors from representative mice from each group. **b** Changes in tumor size. **c** Kaplan–Meier survival curves for CT26 tumor-bearing mice. Statistical significance was calculated by comparison with PBS or *S.t*-ΔpG ^lux/pT-ClyA(+)^+IL-1βAb

*BALB/c* mice transplanted with CT26 tumors were intravenously injected with PBS, ΔppGpp *S. typhimurium* (SL), or ΔppGpp *S. typhimurium* carrying ClyA (*S.t*-ΔpG^lux/pT-ClyA^) to evaluate the antitumor activity of engineered bacteria. Previous research reported tumor recurrence after treatment, which was related to decreased IL-1β levels (Kim et al. 2015). We examined the role of IL-1β in *Salmonella*-mediated cancer therapy by blocking its activity with an anti-IL-1β antibody. As such, a separate group received an intratumoral injection of ΔppGpp *S. typhimurium* carrying ClyA + anti-IL-1β antibody (*S.t*-ΔpG^lux/pT-ClyA^ + IL-1β Ab). We discovered ΔppGpp *S. typhimurium* carrying lux/pT-ClyA exerted the strongest inhibitory effect on tumor growth induced by doxycycline compared to other groups. In contrast, other groups’ tumors regrew at the end of treatment. Tumors injected with PBS grew at the same rate as non-treated tumors. These results indicate that treatment with engineered *Salmonella* expressing lux/pT-ClyA results in significant suppression of tumor growth, and IL-1β production by *S.t*-ΔpG^lux/pT-ClyA^ increases therapeutic efficacy (Fig. 3a, b). In addition, the group of animals that received engineered lux/pT-ClyA-expressing bacteria exhibited higher survival rates than other groups (Fig. 3c).

**Fig. 4:**
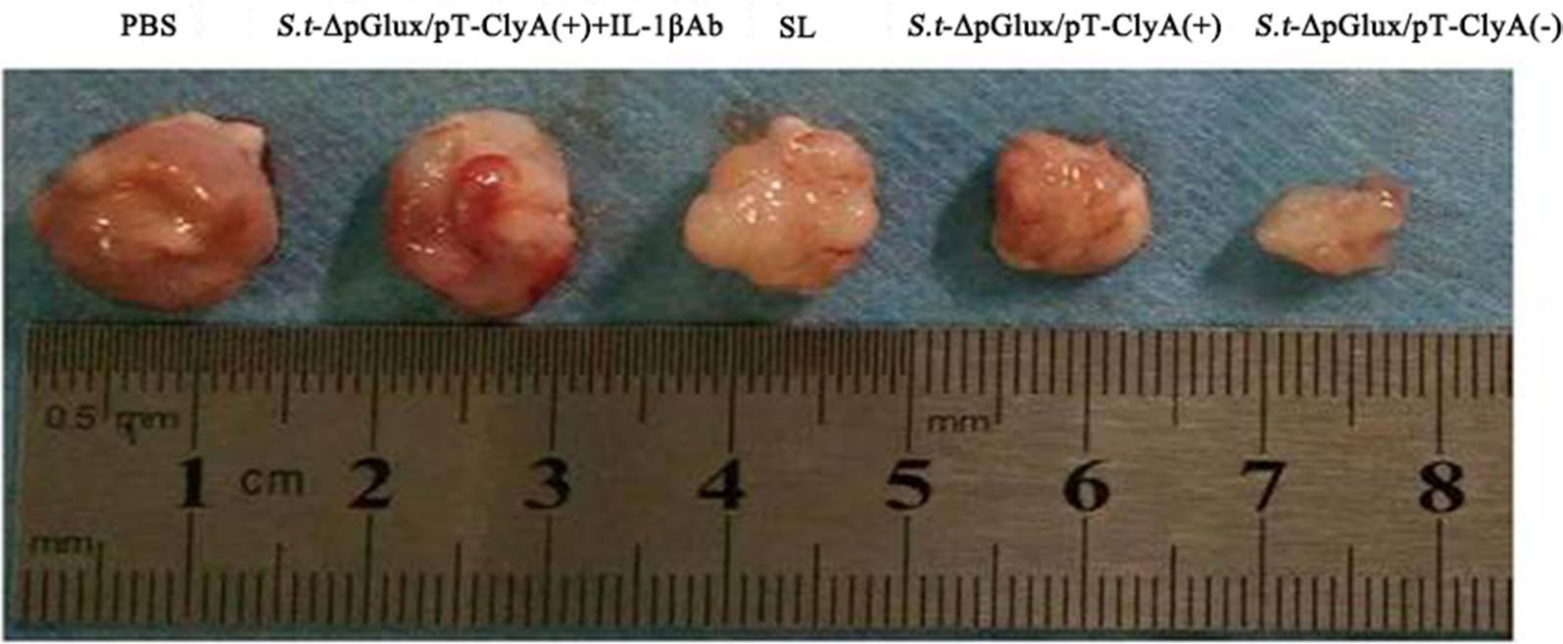
Secreting-lux/pT-ClyA engineered bacteria repress tumor growth in vivo. Tumor volumes of the *S. typhimurium* carrying ClyA (+doxycycline induction) group were smaller compared to other groups

Next, tumors were visualized after dissection. The visual map clearly shows that the tumor volume of mice treated with ΔppGpp *S. typhimurium* carrying ClyA (+Doxycycline induction) group was smaller compared to other groups (Fig. 4).

### Secreting-lux/pT-ClyA engineered *Salmonella* induce elevated IL-1β serum levels

**Fig. 5:**
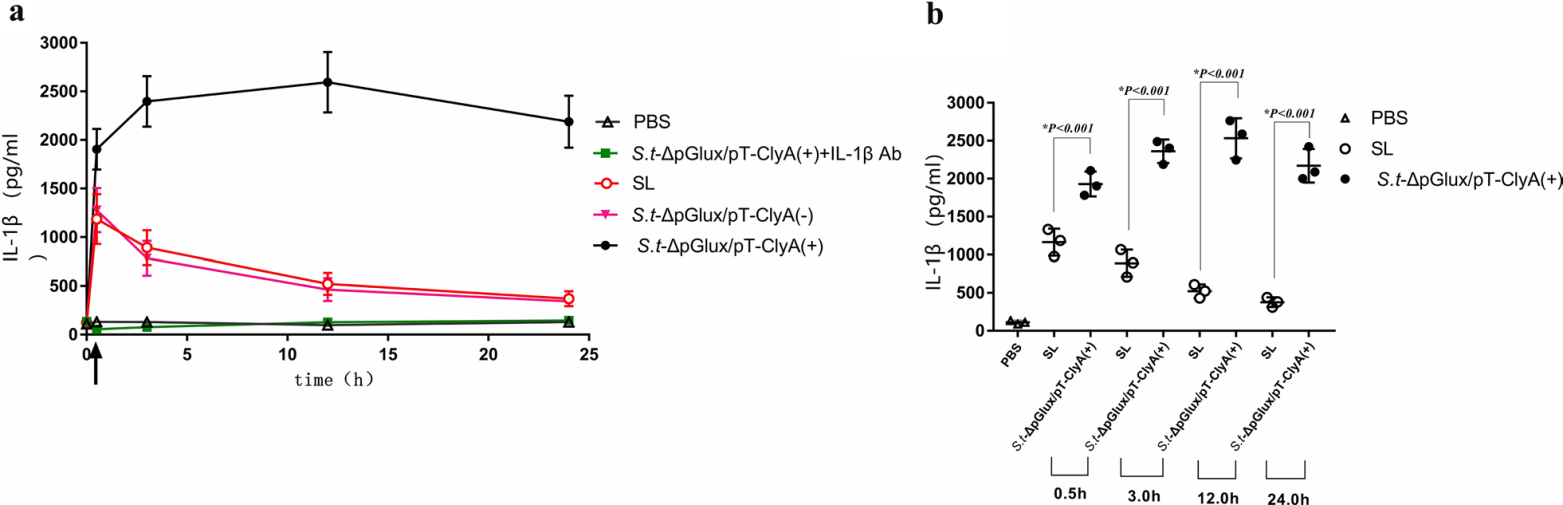
Serum IL-1β levels in different groups at different time points. **a** Serum IL-1β levels were measured by ELISA. Results showed that serum IL-1β levels were significantly higher in mice treated with engineered secreting-lux/pT-ClyA *salmonella* compared to other groups. **b** Serum IL-1β levels in PBS, SL, *S.t*-ΔpG^lux/pT-ClyA(+)^ at indicated times

We examined serum levels of IL-1β in each group. We found that IL-1β serum levels were significantly higher in mice treated with expressing-lux/pT-ClyA *S.t*-ΔpG^lux/pT-ClyA^ than in other groups treated with PBS, SL, SL plux-pT-ClyA(−), SLplux-pT-ClyA(+)+IL-1β Ab, respectively (Fig. 5a). Specifically, we analyzed serum IL-1β levels in PBS, SL, and *S.t*-ΔpG^lux/pT-ClyA(+)^ groups at indicated times (Fig. 5b). Result indicated that secreting-lux/pT-ClyA engineered *Salmonella* increased production of IL-1β in serum.

### Dendritic cells and macrophages are responsible for increased IL-1β levels in tumors colonized by engineered *Salmonella* (S.t-ΔpG^lux/pT-ClyA^)

**Fig. 6:**
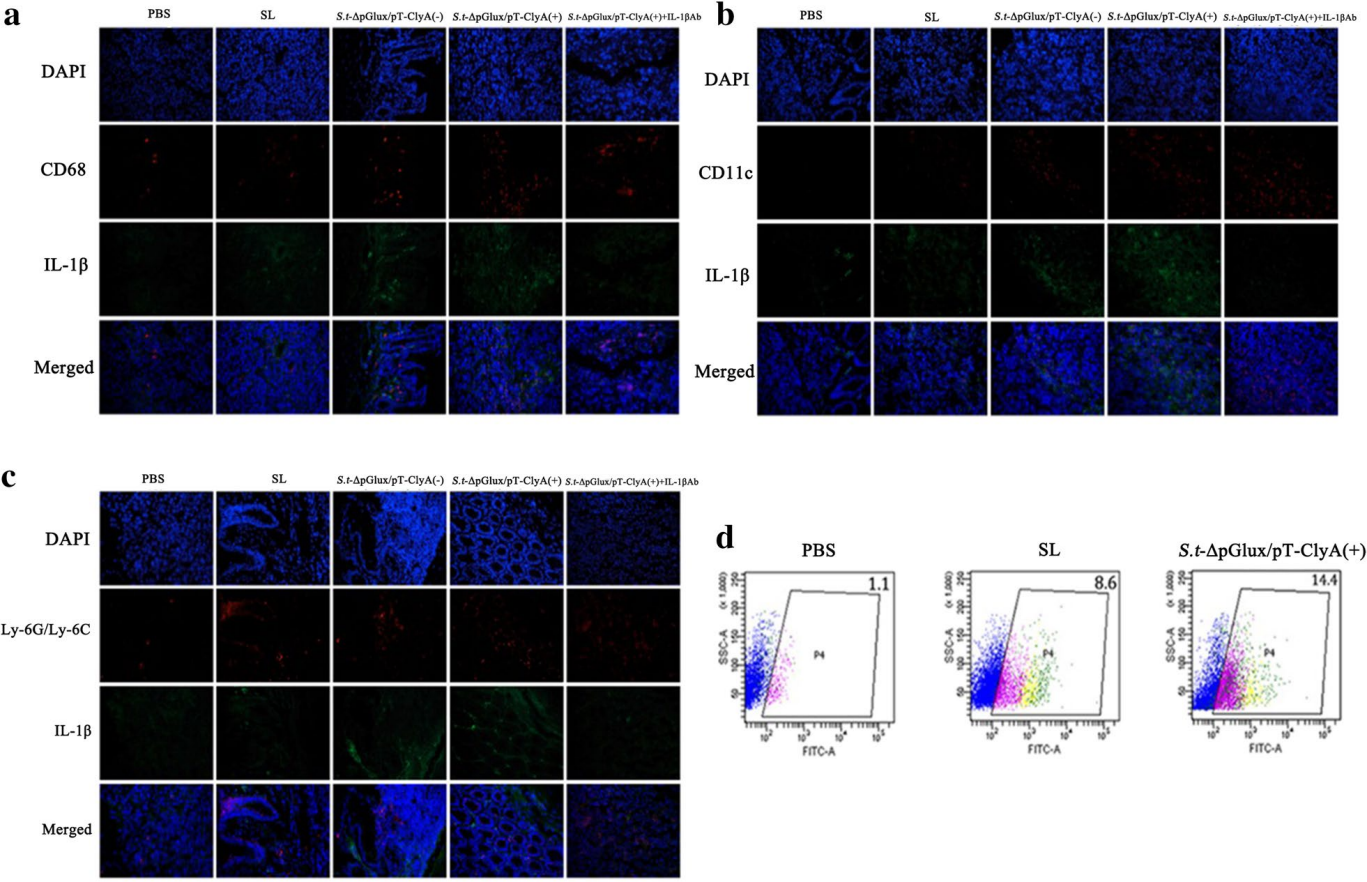
Analysis of immune cell accumulation in tumor tissues and levels of IL-1β. **a**–**c** Tumor tissue was excised and fixed in 3 dpi, cryo-sectioned, and immunostained for IL-1β plus CD68 (macrophages), Ly-6G/ls), or CD11c (dendritic cells). Representative images from three independent experiments are shown. **d** Flow cytometry analysis was used to examine levels of IL-1β in each group in the tumor-draining spleen

Next, we examined immune cell infiltration into tumors colonized by bacteria. Immune cells (macrophages, dendritic cells, and neutrophils) may represent the primary sources of pro-inflammatory IL-1β. We double stained macrophages (CD68), neutrophils (Ly-6G/Ly-6C) and DCs (CD11c) for IL-1β and examined cells under an immunofluorescence (IF) microscope. First, we found that bacterial treatment resulted in increased infiltration of tumor tissues by immune cells. CT26 colon cancer mice intravenously injected with lux/pT-ClyA-secreting engineered *salmonella* exhibited the highest levels of invasive immune cells and IL-1β expression, consistent with previous studies (Kim et al. 2015). Second, some macrophages and DCs (CD11c) colocalized with the IL-1β signal, but neutrophils are only slightly or not colocalize with IL-1β, indicating that DCs and macrophages are the primary producers of IL-1β. Third, CT26 colon cancer mice that received an intratumoral injection of *S.t*-ΔpG ^lux/pT-ClyA(+)^+IL-1β Ab had many infiltrated immune cells, but IL-1β was rarely produced (Fig. 6a–c). Flow cytometry analysis supported the IF microscopic findings, showing that treatment with engineered *Salmonella* led to significant increases in IL-1β expression (Fig. 6d).

### Engineered *Salmonella typhimurium* secreting ClyA enhances cancer immunotherapy by secreting IL-1β through two pathways

**Fig. 7:**
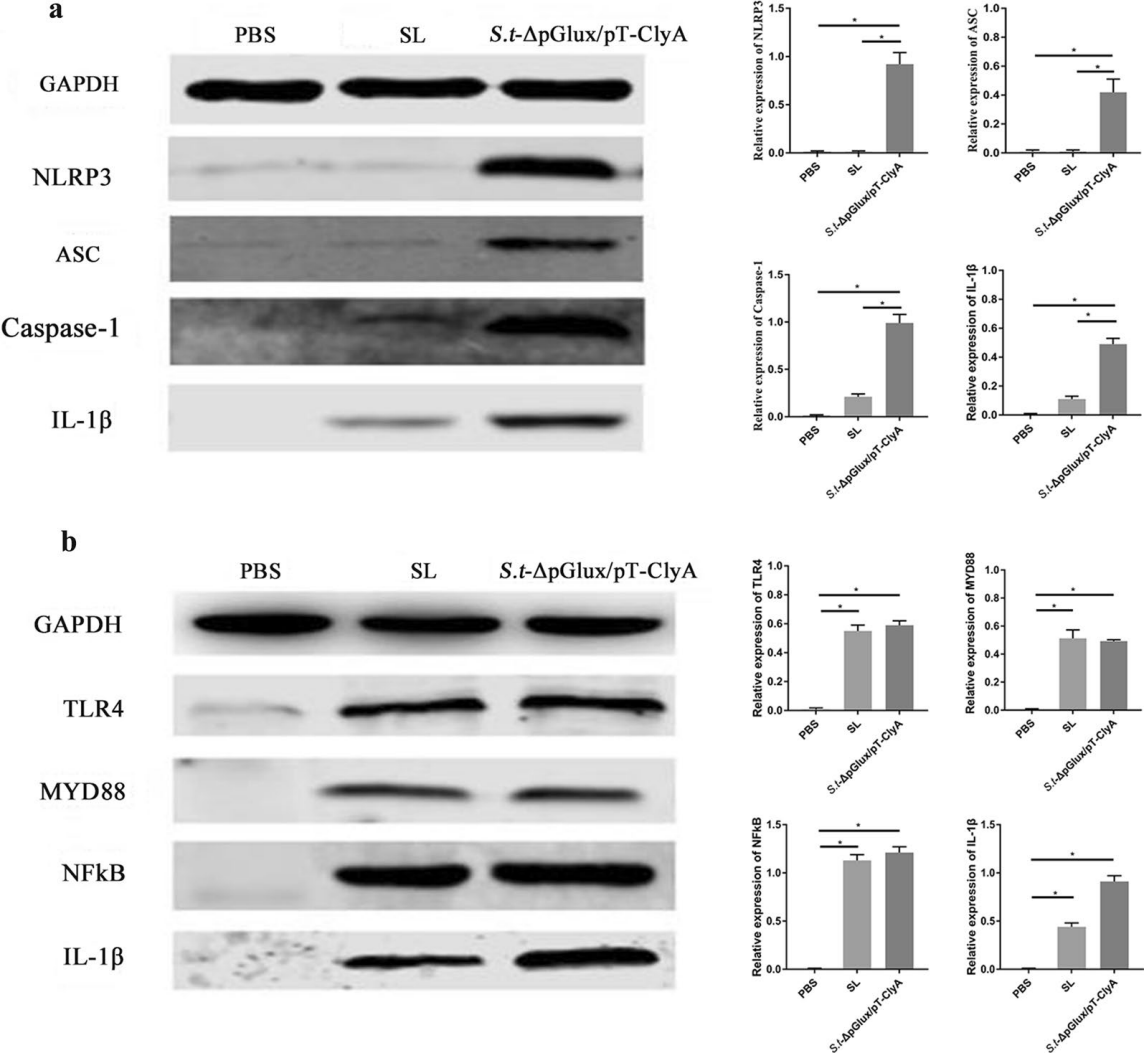
Differences in protein levels of two pathways in different groups of mice. Expression of TLR4, MYD88, NFkB, NLRP3, ASC, Caspase-1, and IL-1β was examined by western blotting. GAPDH was used as a loading control. **a** NLRP3 signaling pathway. **b** TLR4 signaling pathway

To determine whether the antitumor effects of lux/pT-ClyA-secreting ΔppGpp *S. typhimurium* were mediated through host TLR4 and NLRP3 signaling pathways, we investigated expression levels of NLRP3, ASC, caspase1, TLR4, MyD88, and NF-kB in control and experimental group tumor tissues using western blotting (Fig. 7).

Results demonstrated that mice treated with *S.t*-ΔpG^lux/pT-ClyA^ exhibited significant increases in IL-1β was notably increased, and TLR4 and NLRP3 signaling pathways were markedly activated, with their expression levels being significantly higher than other groups. Thus, in the present study, upregulation of IL-1β was observed, indicating that engineered *S. typhimurium* secreting lux/pT-ClyA enhances cancer immunotherapy by inducing IL-1β expression through two pathways.

## Discussion

Lipopolysaccharide (LPS) and cytolysin (ClyA) are important ligands for the activation of toll-like receptor 4 (TLR4) and NOD-like receptor (NLRP3) signaling pathways, respectively (Gupta et al. 2014; Wallace et al. 2000; Mariathasan et al. 2004). IL-1β production is closely correlated with TLR4 and NLRP3 signaling pathways (Phongsisay 2016; Jimenez-Dalmaroni et al. 2016). Moreover, both innate immune cells and colon cancer cells exhibit expression of TLR4 and NLRP3 inflammatory bodies (Basith et al. 2012; Ungerback et al. 2012; Tang et al. 2010; Lee et al. 2012). Activation of the TLR4 signaling pathway leads to activation of extracellular TLR4 via the MyD88-dependent pathway, forming a TLR4 homodimer. Activated TLR4 binds to the C-terminal TIR of the intracytoplasmic junction protein MyD88 via the toll/IL-1 receptor homology region (TIR) in the cytoplasmic region and is recruited by the N-terminal death domain of MyD88 to bind to the IL-1 receptor. The kinase (IRAK) activating the TIR-MyD88/IRAK-NF-kB pathway expresses the inflammatory cytokine IL-1β (Additional file 1: Figure S1) (Shcheblyakov et al. 2010; Mariathasan et al. 2004; Lee et al. 2012). NLRP3 activation of the N-terminal thermoprotein domain (PYD) causes NLRP3 self-oligomerization, which in turn binds to apoptosis-associated microparticle proteins and recruits Pro-caspase1 to form the NLRP3 inflammatory complex (Strowig et al. 2012). Upon activation of the inflammatory complex, Pro-caspase 1 is cleaved to form caspase 1, which then promotes IL-1β release to the extracellular environment. We found that K+ efflux is a necessary signal for NLRP3 activation, and Cytolysin A (ClyA) forms a channel on the cell membrane that causes a large and sustained K+ outflow, activating the NLRP3 pathway and promoting sustained IL-1β release (Wallace et al. 2000) (Additional file 2: Figure S2). Our previous studies successfully transfected the ClyA gene into attenuated *Salmonella* ΔppGpp by genetic engineering technology to form engineered *Salmonella St*-ΔpG^lux/pT-ClyA^ and confirmed that the construct had an obvious inhibitory effect on subcutaneous tumor inoculation of CT26 colorectal cancer (Jiang et al. 2013). We hypothesized that engineered *Salmonella S.t*-ΔpG^lux/pT-ClyA^ acts by activating TLR4 and NLRP3 signaling pathways to promote increased and sustained IL-1β production. In this research, ELISA results showed that colonization of the engineered *Salmonella S.t*-ΔpG^lux/pT-ClyA^ resulted in a significant increase in serum IL-1β levels compared to other groups. At the same time, we also found that in *St*-ΔpG^lux/pT-ClyA^-treated tumors, expression of involved signaling pathway molecules, such as NLRP3, ASC, Caspase1, TLR4, MyD88, NF-kB and IL-1β, were up regulated; however, TLR4, MyD88, NF-kB and IL-1β were up regulated and NLRP3, ASC, and Caspase1 were rarely expressed or not expressed in the ΔppGpp *S. typhimurium* group. These findings confirmed our hypothesis that engineered *Salmonella S.t*-ΔpG^lux/pT-ClyA^ significantly activates TLR4 and NLRP3 signaling pathways, which promotes sustained IL-1β production through two pathways. Phan et al. showed showed highly expression of NLRP3 when treated with this bacterial strain only (Phan et al. 2015), which is different from our results and we will be further demonstrated in the next experiment.

This study demonstrated that IL-1β plays an important role in the anti-tumor process, and IL-1β source cells were primarily monocytes (Hazuda et al. 1990), macrophages (Netea et al. 2009), dendritic cells (Ghiringhelli et al. 2009) and neutrophils (Guma et al. 2009). Dendritic cell-derived IL-1β exerts anti-tumor effects by promoting CD8+ T cells to produce IFN-γ (Ghiringhelli et al. 2009). Recent studies confirmed that tumor cell-derived IL-1β increases the survival rate of EB virus-induced nasopharyngeal carcinoma via recruiting concentrated granulocytes (Chen et al. 2012). However, other studies found that low IL-1β concentrations promote CD4+ T cells to secrete IL-17, inhibiting the body’s anti-tumor effects, while high IL-1β concentrations activate CD8^+^ T cells and promote anti-tumor effects (Ghiringhelli et al. 2009; Bruchard et al. 2013). This suggests that the IL-1β anti-tumor activity is concentration-dependent, and increasing IL-1β concentration will increase its anti-tumor effects. The ΔppGpp *S. typhimurium* lipopolysaccharide (LPS) promoted mononuclear/macrophage and dendritic cells to secrete IL-1β through the TLR4 signaling pathway, exerting anti-cancer effects, but tumor recurrence occurred in late treatment stages, which might be related to reduced IL-1β levels (Kim et al. 2015). Results of this research show that blocking IL-1β activity using an IL-1β antibody abolished the anti-tumor effects of *S.t*-ΔpG^lux/pT-ClyA^. Given these results, IL-1β has potential anti-cancer activity in the context of bacterial-mediated tumor immunotherapy. Treatment with ΔppGpp *S. typhimurium* alone resulted in transient tumor suppression. Then, tumors began to grow again, and IL-1β levels returned to baseline. However, treatment with engineered *Salmonella S.t*-ΔpG^lux/pT-ClyA^ consistently inhibited tumor growth, and IL-1β levels remained high. The survival rate of mice treated with engineered *Salmonella S.t*-ΔpG^lux/pT-ClyA^ was significantly higher than other experimental groups, indicating that the attenuated *Salmonella* LPS and ClyA genes were combined by bioengineering technology to release high levels of IL-1β, inhibit tumor growth and improve survival rate. This overcame the tumor recurrence phenomenon caused by downregulation of IL-1β during late stages of colon cancer treated with ΔppGpp *S. typhimurium* alone.

Immunofluorescence results demonstrated that treatment of tumors with *S.t*-ΔpG^lux/pT-ClyA^ resulted in a significant increase in tumor infiltration by macrophages (CD68), dendritic cells (CD11c), and neutrophils (Ly-6G/Ly-6C). Previous studies revealed that colonization and proliferation of engineered bacteria significantly increased the proportion of M1-like macrophages and reduced the number of Tregs in tumor tissues (Kim et al. 2015). This means that the engineered bacteria inhibited tumor growth by enhancing the anti-tumor immune response rather than by directly inhibiting growth of the tumor itself. Herein, we found that infiltrating tumor cells produced significant levels of IL-1β in response to intravenous injection of engineered bacteria, resulting in inhibition of tumor growth. In addition, we found that immune cells, particularly dendritic cells and macrophages, were the primary sources of IL-1β production in colonized tumors.

In conclusion, *S.t*-ΔpG^lux/pT-ClyA^ showed significant anti-cancer effects in a mouse orthotopic colon cancer model, providing new avenues for colon cancer treatment. LPS and ClyA are important factors in activating TLR4 and NLRP3 signaling pathways, respectively. *St*-ΔpG^lux/pT-ClyA^ activated TLR4-MyD88-NF-Kb-IL-1β and NLRP3-ASC-Caspase-1-IL-1β signaling pathways simultaneously, which enhanced tumor suppressive effects and inducing increased IL-1β release; these results act to overcome the tumor recurrence caused by IL-1β downregulation during late stages of colorectal cancer treatment that are a result of treatment with only ΔppGpp *S. typhimurium*.

## Supplementary information

**Additional file 1: Figure S1.** TLR4 signaling pathway. Activation of the TLR4 signaling pathway leads to activation of extracellular TLR4 via the MyD88-dependent pathway, forming a TLR4 homodimer. Activated TLR4 binds to the C-terminal TIR of the intracytoplasmic junction protein MyD88 via the toll/IL-1 receptor homology region (TIR) in the cytoplasmic region and is recruited by the N-terminal death domain of MyD88 to bind to the IL-1 receptor. The kinase (IRAK) activating the TIR-MyD88/IRAK-NF-kB pathway expresses the inflammatory cytokine IL-1β.

**Additional file 2: Figure S2.** NLRP3 signaling pathway. NLRP3 activation of the N-terminal thermoprotein domain causes NLRP3 self-oligomerization, which in turn binds to apoptosis-associated microparticle proteins and recruits Pro-caspase1 to form the NLRP3 inflammatory complex. Upon activation of the inflammatory complex, Pro-caspase 1 is cleaved to form caspase 1, which then promotes IL-1β release to the extracellular environment. K^+^ efflux is a necessary signal for NLRP3 activation, and Cytolysin A forms a channel on the cell membrane that causes a large and sustained K^+^ outflow, activating the NLRP3 pathway and promoting sustained IL-1β release.

## Data Availability

The data supporting the conclusions of this article are included within the article. Data and materials can also be requested from the corresponding author.
