# Supplementary figures and images for: Secreting-lux/pT-ClyA engineered bacteria suppresses tumor growth via interleukin-1β in two pathways
Source: AMB Express. 2019 Nov 21;9:189. doi: 10.1186/s13568-019-0910-6 (PMC6872689; doi:10.1186/s13568-019-0910-6)

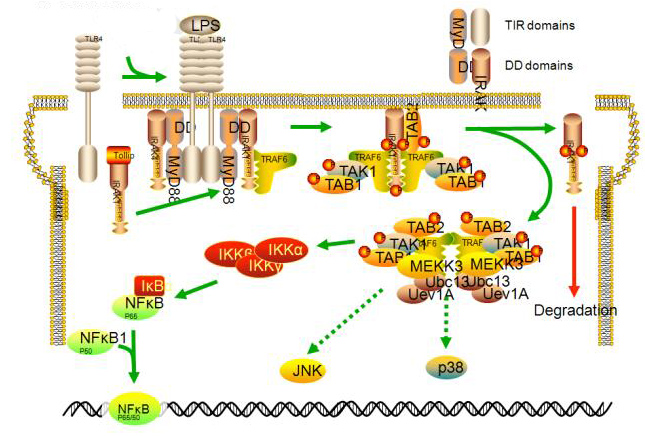

Supplement: Supplementary file 1 — Additional file 1: Figure S1. TLR4 signaling pathway. Activation of the TLR4 signaling pathway leads to activation of extracellular TLR4 via the MyD88-dependent pathway, forming a TLR4 homodimer. Activated TLR4 binds to the C-terminal TIR of the intracytoplasmic junction protein MyD88 via the toll/IL-1 receptor homology region (TIR) in the cytoplasmic region and is recruited by the N-terminal death domain of MyD88 to bind to the IL-1 receptor. The kinase (IRAK) activating the TIR-MyD88/IRAK-NF-kB pathway expresses the inflammatory cytokine IL-1β. [file 13568_2019_910_MOESM1_ESM.jpg]

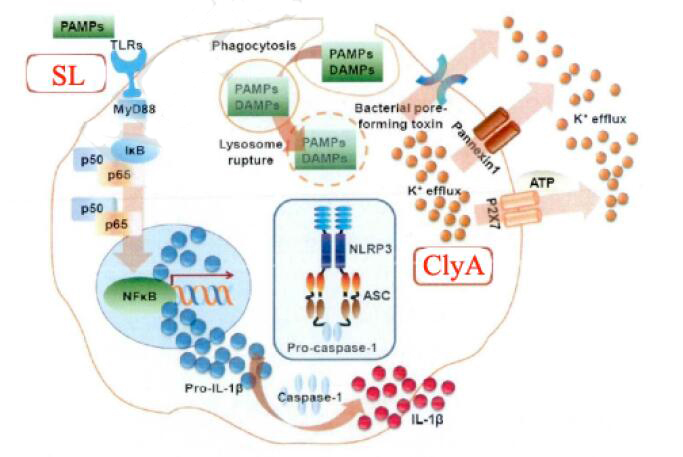

Supplement: Supplementary file 2 — Additional file 2: Figure S2. NLRP3 signaling pathway. NLRP3 activation of the N-terminal thermoprotein domain causes NLRP3 self-oligomerization, which in turn binds to apoptosis-associated microparticle proteins and recruits Pro-caspase1 to form the NLRP3 inflammatory complex. Upon activation of the inflammatory complex, Pro-caspase 1 is cleaved to form caspase 1, which then promotes IL-1β release to the extracellular environment. K+ efflux is a necessary signal for NLRP3 activation, and Cytolysin A forms a channel on the cell membrane that causes a large and sustained K+ outflow, activating the NLRP3 pathway and promoting sustained IL-1β release. [file 13568_2019_910_MOESM2_ESM.jpg]
